# High Intensity Interval Training in Handcycling: The Effects of a 7 Week Training Intervention in Able-bodied Men

**DOI:** 10.3389/fphys.2016.00638

**Published:** 2016-12-23

**Authors:** Patrick Schoenmakers, Kate Reed, Luc Van Der Woude, Florentina J. Hettinga

**Affiliations:** ^1^Centre of Sport and Exercise Sciences, School of Biological Sciences, University of EssexColchester, UK; ^2^Center for Human Movement Sciences and Centre for Rehabilitation, University Medical Centre Groningen, University of GroningenGroningen, Netherlands

**Keywords:** upper body exercise, endurance training, handcycling, physiological capacity

## Abstract

**Introduction:** In lower body endurance training, quantities of both moderate intensity continuous training (MICT) and high intensity interval training (HIIT) can lead to an improved physiological capacity and performance. Limited research is available regarding the endurance and muscular capacity of the upper body, and how training contributes to improvements in performance capacity is still unknown. The aim of the current study was to evaluate the effects of HIIT and MICT on the physiological capacity and handcycling performance of able-bodied men in a well-controlled laboratory setting.

**Methods:** Twenty four recreationally active men (22 ± 2 years; 1.84 ± 0.04 m; 79 ± 10 kg) were matched on incremental handcycling pre-test performance (peakPO) and then randomly assigned to HIIT, MICT, or a non-training control group (CON, 3 × *n* = 8). Participants in HIIT completed 14 interval training sessions, performing 4 × 4 min intervals at 85% heart rate reserve (%HRR), and seven continuous training sessions at 55 %HRR (every 2nd training session of the week). Participants in MICT performed 21 training sessions of 30 min at 55 %HRR. After the intervention, changes in peak oxygen uptake (peakVO_2_) and peak power output (peakPO) were compared within and between HIIT, MICT and CON.

**Results:** The average external training load per training session did not differ between MICT and HIIT (*p* = 0.713). Improvements after HIIT in peakVO_2_ (22.2 ± 8.1%) and peakPO (47.1 ± 20.7%) were significantly larger compared with MICT and CON (*p* < 0.001). Improvements after MICT in peakVO_2_ (10.7 ± 12.9%) and peakPO (32.2 ± 8.1%) were higher compared to CON (*p* < 0.001). Higher improvement after HIIT occurred despite training 22% less time than MICT. No significant changes were found in CON.

**Discussion:** As in lower body endurance sports, HIIT proved to be very effective in improving the physiological and performance capacity of upper body exercise. Whilst physiological capacity in both training groups improved significantly compared with CON, the present study shows that peakVO_2_ and peakPO improved more after HIIT than after MICT in able-bodied men. It is advised to include HIIT into training regimes of recreational and competitive handcyclists to improve the upper body endurance capacity.

## Introduction

Endurance performance is regulated by, amongst other factors, the cardiovascular system, the pulmonary system, the lactate metabolism and the exercise economy of an athlete (Joyner and Coyle, 2008). The essence of training is to provide training loads that are effective in improving the performance capacity of athletes. Adaptations to endurance training are well documented for lower body exercise like running and cycling, in which doses of both moderate intensity continuous training (MICT) and high intensity interval training (HIIT) result in increases in the physiological and performance capacities of endurance athletes (Laursen, 2010; Buchheit and Laursen, 2013). Although these training modalities stimulate mitochondrial biogenesis differently (Gibala and McGee, 2008; Laursen, 2010), both training techniques result in an increased capacity to generate ATP aerobically, which ultimately can lead to an increased endurance performance.

When athletes are dependent on their upper body in endurance events—for example in handcycling or wheelchair racing—less active muscle mass is available to generate power compared to lower body exercise. It is proposed that training a smaller muscle mass may result in different physiological responses to endurance training compared to exercise regimes involving the larger lower bodies' muscle mass (Miles et al., 1989; Schneider et al., 2002). To date, limited research is available regarding upper body endurance training and the accompanying physiological adaptations. In previous studies, different MICT handcycling and armcranking protocols resulted in improved peak oxygen uptake (peakVO_2_) and peak power output (peakPO) in disabled and/or spinal cord injured patients, in healthy elderly and in able-bodied men and women (Franklin, 1989; Pogliaghi et al., 2006; Valent et al., 2007; Hettinga et al., 2016). In lower body endurance training, HIIT was shown to be a time-efficient training method to induce both central and peripheral adaptations (Gibala et al., 2012), and is now considered more effective at improving the physiological and performance capacity of untrained individuals, recreationally active and trained athletes compared to MICT for a given training volume (Weston et al., 2014; Milanović et al., 2015). The effects of HIIT in upper body endurance training have been studied in patients with chronic tetraplegia (Valent et al., 2009). Although significant improvements in peakVO_2_ and peakPO were reported in this study (Valent et al., 2009), the findings are of limited value in the context of sports training, due to large respiratory, biomechanical and metabolic differences between patients undergoing active rehabilitation and trained handcyclists (Lovell et al., 2012; Fischer et al., 2014; de Groot et al., 2014). In order to develop understanding and guidelines for upper body endurance training for sports and/or advanced rehabilitation practice, the effectiveness and possible role of HIIT is yet to be established and compared with other training protocols.

The aim of the current study was to evaluate the effects of HIIT, known to be effective in lower body exercise, and MICT, a protocol more common in handcycling, on the physiological capacity and handcycling performance of able-bodied men in a well-controlled laboratory setting. It was hypothesized that HIIT would improve peakVO_2_ and peakPO, but not to a larger extent than a period of MICT. A secondary aim of this study was to add to the scarce reference data available concerning able-bodied participants during handcycling. Reference data are necessary to better understand the physiology of the upper body in relation to exercise using handcycling, to facilitate the interpretation of data from disabled individuals.

## Methods

### Participants

Twenty four recreationally active able-bodied men, unaccustomed to upper body endurance training, volunteered to take part in the study (mean ± SD: age: 22 ± 2 year; height: 1.84 ± 0.04 m; body mass: 79 ± 10 kg). During an initial visit, study details and participation requirements were explained, and written informed consent was obtained. During visit one, participants completed three 6 min bouts of handcycling on a motor driven treadmill to familiarize with the handcycle's propulsion and steering mechanisms. Participants were instructed not to alter other training activities outside those of the study protocol. The study received approval from the local ethics committee (Center for Human Movement Sciences, Groningen) and was conducted in accordance with the Declaration of Helsinki.

### Design

Before and after the 7-week training intervention, an incremental handcycling test was performed to obtain peak cardiovascular variables and to evaluate handcycling performance. Based on peakPO ascertained during the incremental pre-test, participants were matched and then randomly assigned to HIIT, MICT or a non-training control group [(CON), 3 × *n* = 8]. Participants in HIIT and MICT visited the laboratory three times per week for 7 weeks to complete a total of 21 training sessions. Participants in HIIT completed 2 interval training sessions per week, performing 4 × 4 min intervals at 85% heart rate reserve (%HRR), and 1 moderate intensity continuous training sessions at 55 %HRR (every 2nd training session of the week). The MICT group performed 3 continuous training sessions of 30 min per week, at an average training intensity of 55 %HRR. CON received no training, and was asked to maintain their regular activity level during the experimental period.

**Figure 1:**
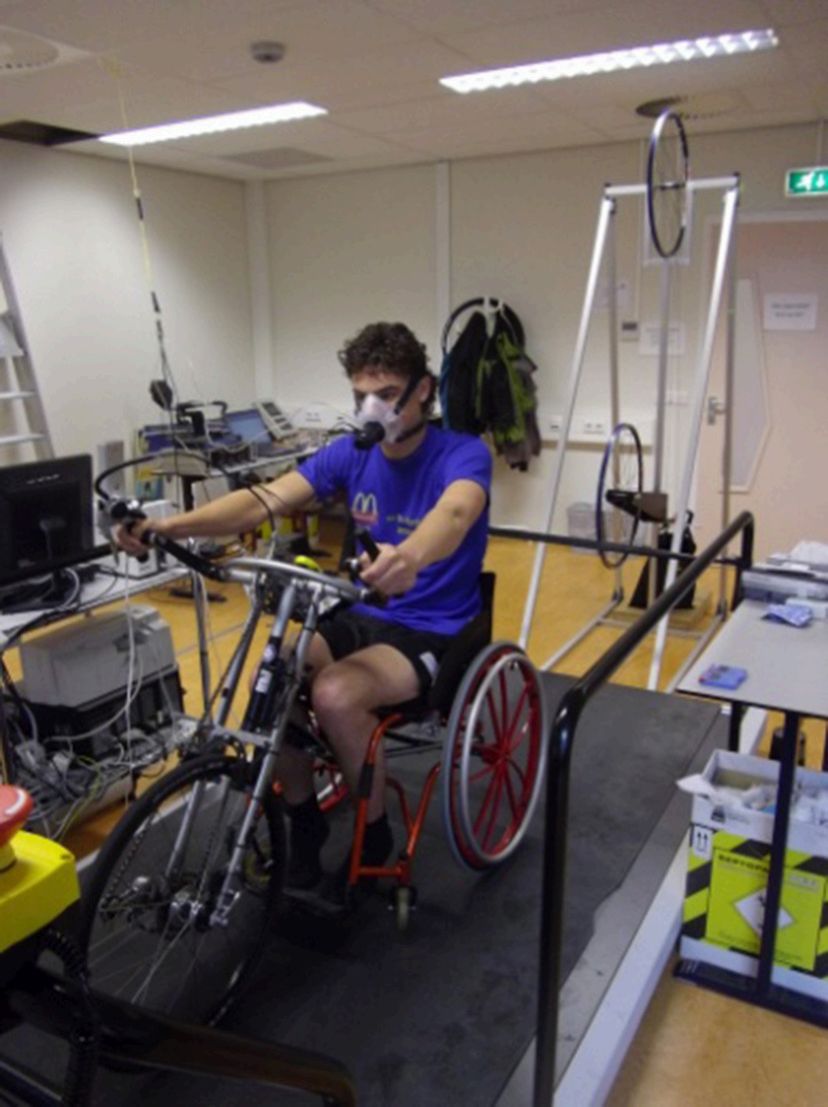
**The experimental handcycle, consisting of a wheelchair with a mounted handcycling unit in front of the pulley system on the motor driven treadmill**.

All training sessions and incremental tests were performed on a motor driven treadmill (Enraf Nonius, The Netherlands) in a handcycle (see Figure 1), which consisted of a wheelchair (RGKWheelchair Inc., England) with a mounted handcycling unit (Double Performance, The Netherlands). Prior to each training session, participants in both HIIT and MICT performed 2 × 4 min self-paced warm up bouts on a customized handcycle (Wolturnus, Denmark) placed on a cycletrainer (Tacx B.V., The Netherlands). Training sessions were monitored by heart rate (Polar Electro, Finland) and power output (PowerTap SL, United States). After each training session rating of perceived exertion (RPE, Borg, 1970) and local perceived discomfort (LPD) of the upper body were obtained (Bafghi et al., 2008).

### HIIT training

**Figure 2:**
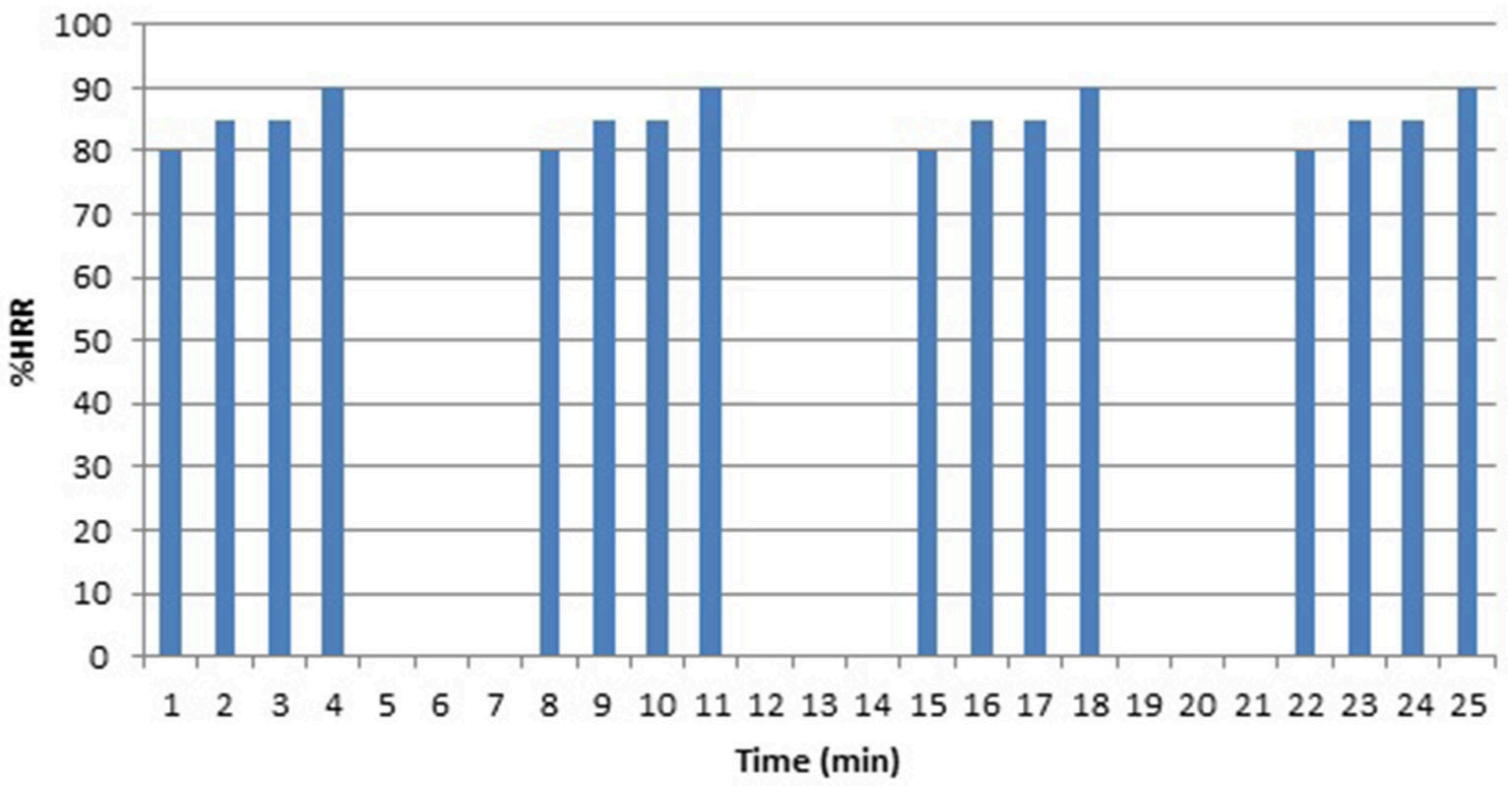
**Temporal pattern (as %HRR per minute) that was imposed in the 4 × 4 min interval training sessions in HIIT, in which the resistance was changed after every minute in the 4 min work intervals**.

In the first and third training session of each week, HIIT participants completed a 4 × 4 min interval training protocol. During the 4 min work intervals, average training intensity was 85 %HRR (see Figure 2), based on a protocol previously used by Helgerud et al. (2007) in untrained runners. Exercise intensity was achieved by adding or reducing the workload through the pulley system placed behind the treadmill (as described in Hettinga et al., 2016, see Figure 1), while riding at a fixed velocity of 1.67 m·s^−1^. Workload was adjusted after every minute in the 4-min work intervals. Between work intervals, participants received 3 min of passive rest. In the first 2 weeks of the training program, training intensity in the work intervals increased from 65 to 85 %HRR to minimize the risk of injuries and overtraining. The second training session of each week was a continuous training session of 30 min, as described in MICT Training below.

### MICT training

**Figure 3:**
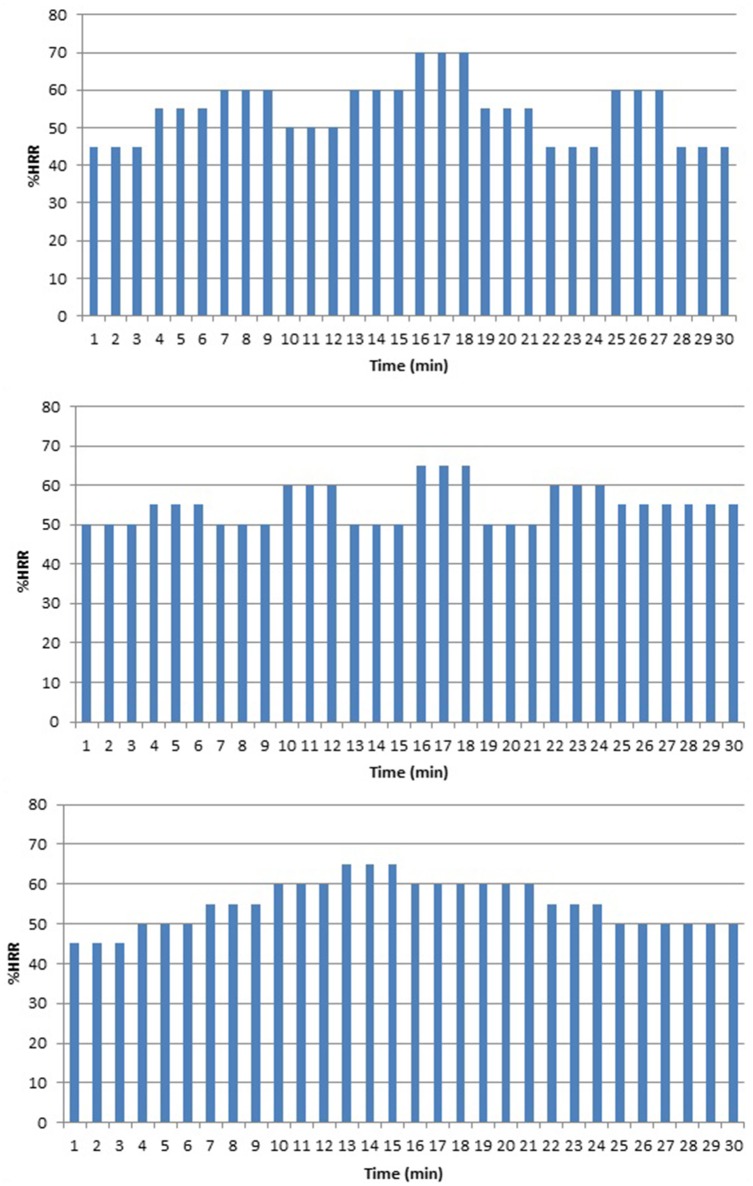
**Temporal patterns (as %HRR per minute) that were imposed in the 30 min continuous training sessions in MICT and HIIT by varying either velocity or resistance after every 3 min**.

In MICT, participants performed three continuous training sessions per week, each for a duration of 30 min at an average exercise intensity of 55 %HRR. This intensity was achieved by either “resistance” or “velocity” training, as is common in wheelchair (van der Woude et al., 1999) and handcycle training (Hettinga et al., 2016). Three different temporal patterns were used in a fixed sequence for each subject (see Figure 3), to vary the training stimulus systematically over time (van der Woude et al., 1999). In resistance training, the workload was varied around a mean exercise intensity of 55 %HRR by adding or reducing workload through the pulley system every 3 min, while the velocity was kept constant at 1.67 m·s^−1^. During velocity training, resistance was kept constant at a workload corresponding to the workload required to handcycle at 55 %HRR, only now velocity was varied every 3 min. To assure a comfortable cadence (50–90 rpm) gearing was changed on increased riding velocities. MICT performed 11 “velocity” sessions and 10 “resistance” over the course of the study.

### Incremental exercise test

Participants performed an incremental exercise test before and after the training intervention. Participants were asked to refrain from consuming alcohol and caffeine for at least 24-h, as well as from engaging in strenuous exercise for at least 48-h prior to testing. Both incremental pre-test and post-tests were performed at the same time of the day, to minimize circadian effects. To elicit valid maximal physiological values for upper body exercise, a test protocol was designed specific to this group after several pilot tests conducted in our laboratory. In the current protocol, gearing was fixed and treadmill velocity was a constant 1.67 m·s^−1^, so participants rode at 70 rpm (Sawka et al., 1983). Before the start of the incremental exercise test, a 5 min submaximal warm up was executed at 30 W. The initial power output of the test was 30 W, which increased 10 W every minute until exhaustion. PO was increased by adding weight to a pulley system at the back of the handcycle (see Figure 1) as introduced by van der Woude et al. (1999). When voluntary exhaustion was reached, or when rpm dropped below 70, the test was ended. Respiratory parameters were measured breath by breath, using open circuit spirometry (Oxycon Delta, Germany). The gas analyzer was calibrated prior to each test using room air, a Jaeger 3-1 syringe and a calibration gas (16.0% O_2_, 5.0% CO_2_). Peak power output (peakPO), peak oxygen uptake (peakVO_2_), peak heart rate (peakHR), peak minute ventilation (peakVE) and the respiratory exchange ratio (RER) were calculated between the 20th and 50th s of every completed minute.

### Data analysis

All data were analyzed and calculated using SPSS 17.0 (SPSS Inc., USA), Office Excel 2010 (Microsoft Corporation, USA) and Matlab 2013 (The Mathworks, USA) and are presented as mean ± SD. Participant and training characteristics were compared using one way ANOVA. The effect of the intervention period on the physiological capacity (peakVO_2_, peakVE, peakHR, and RER) and handcycling performance (peakPO) within and between groups was tested using a repeated measures ANOVA. *Post hoc* Bonferroni pairwise comparisons were used to show differences between experimental groups. The significance level of all tests was set at *p* < 0.05.

## Results

All 24 participants completed the study. At baseline, there were no statistical differences between the three groups with regard to age, height, body mass, and performance or cardiorespiratory variables (see Tables 1, 2). In HIIT, training intensity increased from 65 to 85 %HRR in the first four interval training sessions. Thereafter, 10 interval training sessions were performed at an average intensity of 84.0 ± 1.8 %HRR. The HIIT group performed seven (3 resistance, 4 velocity) continuous training sessions at 55.5 ± 1.4 %HRR. Training intensity for the MICT group during the 21 training sessions averaged 55.3 ± 0.3% HRR. The external training load, calculated as average PO per training session*duration of a session, did not differ between MICT and HIIT [2.32 ± 0.3 kJ vs. 2.28 ± 0.3 kJ, respectively (*p* = 0.713)]. Total training time was significantly lower in HIIT compared to MICT (434 vs. 630 min). HIIT was perceived more exhaustive than MICT (*p* < 0.001), with an average session RPE of 16 ± 1, where MICT was rated 13 ± 2. The HIIT group experienced significantly more discomfort in the upper body during the training, indicated by a LPD score of 10.7 ± 5 compared to MICT who reported on average of 5.9 ± 3 (*p* < 0.001).

**Table 1:** **Participant characteristics for CON, MICT, and HIIT**.

	**CON (*n* = 8)**	**MICT (*n* = 8)**	**HIIT (*n* = 8)**
Age (years)	23 ± 1.7	21 ± 2.3	23 ± 1.8
Height (m)	1.86 ± 0.02	1.83.0 ± 0.04	1.85 ± 0.04
Body mass (kg)	86.6 ± 10.1	75.1 ± 10.3	77.4 ± 7.2

*Data are reported as mean ± SD*.

*No significant differences were found in baseline values between the training groups and the non-training control group*.

**Table 2:** **Changes in physiological capacity and handcycling performance from pre- to post-training period for CON, MICT, and HIIT**.

	**CON**		**MICT**		**HIIT**		
	**Pre-test**	**Post-test**	**Pre-test**	**Post-test**	**Pre-test**	**Post-test**	Interaction (group^*^time)
peakPO (W)	143.3 ± 13.7	143.2 ± 10.4	128.9 ± 26.9	169.0 ± 27.8^*^^§^	133.2 ± 26.2	191.3 ± 16.2^*^^§^	*F*_(2)_ = 72.19, *p* < 0.001
peakVO_2_(ml·kg^−1^·min^−1^)	31.5 ± 3.0	32.9 ± 4.3	33.2 ± 4.5	36.5 ± 4.5^*^^§^	34.3 ± 3.8	41.9 ± 4.9^*^^§^	*F*_(2)_ = 7.66, *p* = 0.003
peakVE (L·min^−1^)	105.7 ± 11.0	104.1 ± 16.2	89.7 ± 20.3	109.4 ± 13.4^*^^§^	99.7 ± 20.1	130.4 ± 13.9^*^^§^	*F*_(2)_ = 11.32, *p* = 0.001
RER	1.24 ± 0.08	1.16 ± 0.06^*^	1.17 ± 0.05	1.24 ± 0.03^*^	1.19 ± 0.05	1.21 ± 0.06	*F*_(2)_ = 12.57, *p* < 0.000
peakHR (bpm)	182 ± 8	186 ± 13	180 ± 21	176 ± 18	188 ± 9	190 ± 3	*F*_(2)_ = 2.25, *p* = 0.132

*Data are reported as mean ± SD*.

*No significant differences were found in baseline values between the training groups and the non-training control group*.

* *Significant different from pre-test (p < 0.05)*.

§ Significant interaction of group^*^time (p < 0.05).

Table 2 shows the peak physiological and performance capacity of both training groups and the non-training control group before (pre) and after (post) the experimental period. Repeated measures ANOVA showed there were significant increases in peakVO2, peakPO and peakVE, both within and between groups over the course of the study (*P* < 0.05). There were no main effects of time or group on body mass, RER, or peakHR.

Interaction effects showed different responses according to group. HIIT resulted in a significantly higher final peakPO than both MICT and CON (*p* < 0.001). The improvement after MICT was significantly higher than CON (*p* < 0.001). These final peakPO values represent a 47.1 ± 20.7% increase for HIIT, a 32.2 ± 8.1% increase for MICT and a 0.3 ± 5.7% increase in CON, from baseline. HIIT resulted in a significantly higher final peakVO_2_ than MICT and CON (*p* < 0.001). The improvement after MICT was significantly higher than after CON (*p* < 0.001). The final peakVO_2_ values represent a 22.2 ± 8.1% increase for HIIT, a 10.7 ± 12.9% increase for MICT and a 4.7 ± 10.2% increase for CON. HIIT (*p* = 0.002) and MICT (*p* = 0.028) resulted in a significantly higher final peakVE than CON. These final peakVE values represent a 34.0 ± 20.6% increase for HIIT, a 25.0 ± 18.3% increase for MICT and a 1.1 ± 13.2% decrease for CON. A significant increase in RER (6.1 ± 2.4%) was found on the post-test for MICT (*p* = 0.002), which was in contrast to the decrease of 6.3 ± 6.0% after CON (*p* = 0.009).

## Discussion

Adaptations to endurance training are well documented for lower body exercise such as running and cycling. However, less research is available regarding upper body endurance training in general, and in handcycling specifically. This study aimed to evaluate the effects of HIIT and MICT on the physiological capacity and handcycling performance of able-bodied men. In lower body exercise, both these training modalities have been shown to improve endurance performance. The most striking outcomes of the present study were the large improvements in peakVO_2_, peakPO, and peakVE after 7 weeks of HIIT. MICT also resulted in notable but smaller improvements in peakVO_2_, peakPO, and peakVE. Thus, it seems that successful training protocols of lower body exercise can be used to design upper body endurance training programs in handcycling.

To date, research on handcycling is scarce and primarily focuses on the use of handcycling in the rehabilitation of spinal cord injured patients (Valent et al., 2009; Hettinga et al., 2010). With the increased interest in handcycling as a competitive sport over the past two decades, scientific interest also increased, resulting in several descriptive studies of the physiological and performance profiles of trained handcyclists (Abel et al., 2006; Lovell et al., 2012; Fischer et al., 2014). In handcycling, athletes compete in mass start road races and time trials in five different ability classes, based on their anatomic level of spinal cord injury and/or associated functional limitations (UCI, 2016). Recently, peakVO_2_ and peakPO were identified as important predictors of time trial (Lovell et al., 2012; de Groot et al., 2014) and race performance in trained handcyclists (Janssen et al., 2001; Fischer et al., 2015). In the present study, after HIIT and MICT there was an increase in both these variables in able-bodied men, indicating the effectiveness and importance of both these training modalities in the design of an optimal endurance training program for upper body exercise, thereby indicating the potential relevance to handcycling athletes and/or patients in an advance rehabilitation setting.

The present study provides interesting insights into the responses to upper body endurance training. The HIIT protocol resulted in significantly higher improvements in peakVO_2_ and peakPO than both MICT and CON after the 7-week intervention period. This despite a 22% lower training time compared to MICT. Although the external training load was not matched *per se* prior to the training interventions, no differences were found in the average training load over the 21 training sessions across training groups. It has been suggested a longer duration of training sessions could compensate for lower intensity exercise (Overend et al., 1992; Warburton et al., 2004). However, the present study, with matched total workload and number of training sessions, does not support this claim. Instead, our results are consistent with those of Helgerud et al. (2007) who found that intensity of training cannot be compensated for by longer duration, and showed larger improvements after HIIT compared to MICT.

In the current study, we adopted an interval training protocol that resulted in an improved VO_2_max(+7.2%) in a group of untrained runners after an 8 week training program (Helgerud et al., 2007). Improvements in HIIT in the current study were substantially larger compared to these findings, which can be explained by the initial inexperience of upper body endurance training in our participants. Osawa et al. (2014) reported an increase in peakPO after a 16-week period of combined leg and arm cranking HIIT. In their study, participants performed 32 training sessions including four 6 × 1 min work intervals at a workload >90% of peakPO, interspersed with 1 min active recovery (Osawa et al., 2014). Given the smaller increase of peakPO along with lower baseline values compared with the current study [96 ± 12 W (+25.0 ± 8.3%) vs. 133.2 ± 26.2 W (+47.1 ± 20.7%)], our interval protocol appears to be favorable to improve handcycling performance. The duration of work intervals is important in the programming of HIIT. Longer duration intervals may be more effective in upper body training, due to the different oxygen uptake kinetics of upper body compared to lower body exercise (Koppo et al., 2002). The relatively slow response of the “fast component” of VO_2_, and the relatively late emergence of the slow VO_2_ component in upper body compared to lower body exercise (Koppo et al., 2002), suggests that the interval duration of HIIT required to improve oxygen uptake must be at least 2 min in order to allow VO_2_ to peak. This is in line with Midgley et al. (2006), who stated that in lower body HIIT, longer work intervals elicit maximal oxygen uptake, or at least a very high percentage of peakVO_2_ and therefore provide a more effective stimulus for enhancing oxygen uptake compared to short duration intervals.

In many lower body endurance sports, around ~80% of athletes' training sessions are performed at a relatively low intensity (Seiler, 2010). The improvements as a result of MICT therefore are of interest in the context of upper body endurance training. The present study showed that a training dose of 7 weeks, 3 × 30 min per week of handcycling at an average of 55 %HRR, resulted in improvements in incremental handcycling performance on the parameters peakVO_2_ and peakPO. The increase in peakPO is in line with the findings of Hettinga et al. (2016), who reported an increase in peakPO after 7 weeks of 3 × 30 min continuous handcycling training at 65 %HRR in able-bodied women. The increase in peakVO_2_ in the current study was lower than the reported increase in the study of Hettinga et al. (2016) (+18.1% vs. 10.7 ± 12.9% respectively). This difference may be explained by the higher baseline values of the male participants (33.2 ± 4.5 ml·kg^−1^·min^−1^) in the current study compared to female participants (28.3 ± 5.1 ml·kg^−1^·min^−1^) in Hettinga et al. (2016). Another explanation for the difference in improvement can be attributed to the difference in relative training intensity. In the current study, participants in MICT trained at an average power output corresponding to 55 %HRR. This training intensity was based on the findings of Knechtle et al. (2004) who reported the highest fat oxidation at 55% peakVO_2_ in well-trained handcyclists. In contrast, the participants in the study by Hettinga et al. (2016) trained at an average power output corresponding to 65 %HRR. Åstrand (2003) stated that the minimum training intensity to improve peakVO_2_ must be around 55–65 %HRR. The limited data now available on handcycling suggest that an exercise intensity of 65 %HRR in MICT is more favorable to improve the upper body endurance capacity.

Both HIIT and MICT improved handcycling performance and physiological capacity. Previous work from our lab (Hettinga et al., 2016) showed that training adaptations after MICT in handcycling are local and exercise specific, since no transfer effects were found in an incremental cycling test. We therefore assume that the adaptations that are responsible for the changes in peakVO_2_ and peakPO after MICT in the current study are primarily local. Based on the results of the current study, it is hard to state which mechanisms are responsible for the differences in improvements after HIIT and MICT. It can be proposed that participants in HIIT became more familiar with higher workloads, as HIIT required higher work intensities. This may have contributed to an increased skeletal muscle buffering capacity, as was apparent in well trained cyclists after 6 HIIT training sessions (Weston et al., 1997). We can also speculate that the higher workloads in HIIT resulted in an increased force of the working muscles in the handcycling motion. The m. deltoid, m. triceps and m. trapezius are the muscles that produce the main force throughout propulsion in handcycling (Arnet et al., 2012). An increase in force of these muscle groups would decrease the relative force in each propulsion at a given submaximal work intensity. This would allow an increased recruitment of the slow twitch (type 1) fibers and a reduced rate of fast twitch (type 2) fiber recruitment. This in turn may result in an improved work efficiency during the (sub)maximal workloads in the incremental handcycling test. Jacobs (2009) showed that 12 weeks of upper body strength training, without any endurance training, increased peakVO_2_in paraplegic individuals. Similarly, the use of heavy strength training has been shown to increase upper body endurance capacity in kayaking (Ualí et al., 2012) and wheelchair racing (Turbanski and Schmidtbleicher, 2010). However, at present this theory is largely speculative in relation to the current study. We did not assess changes in muscle strength after HIIT or MICT, which is a limitation and we are consequently unable to ascertain the relationship between strength, peakPO and peakVO_2_. Adaptations in muscle strength after HIIT and MICT, but also after structured strength training and concurrent endurance training in relation to improvements in handcycling performance should be assessed in future research to determine optimal training regimes.

Previously, the use of an 8–12 week interval training protocol was effective in improving peakVO_2_ and peakPO in the rehabilitation of spinal cord injured patients (Valent et al., 2009). Our study is one of the first to address different training modalities in upper body endurance training. The use of a homogeneous group of able-bodied men allowed us to compare responses to HIIT and MICT in a controlled setting, adding to data available required for establishing training prescriptions for upper body exercise. Although the participants in the current study were not trained handcyclists, average peakVO_2_ and peakPO in this group of able–bodied men was similar to those reported for trained handcyclists (Janssen et al., 2001; de Groot et al., 2014). Based on the results of the current study, it is therefore expected that both HIIT and MICT can alter the physical capacity of recreationally active and trained handcyclists. However, as differences in physiology have been evidenced between able-bodied and disabled individuals (Bernard et al., 2000; Schilero et al., 2009), it is important to evaluate how data collected in able-bodied participants compares with people with different disabilities. The handcycle used in the current study is typically used for activities of daily living (Hettinga et al., 2010). Differences between the experimental handcycle and racing handcycles, the accompanying differences in body positioning, muscle recruitment, and movement possibilities are noteworthy (Zipfel et al., 2009). How these differences might influence adaptations to HIIT and MICT are to be assessed in future research.

## Conclusion

The aim of the current study was to evaluate the effects of a 7 week HIIT or MICT training intervention on the physiological capacity and handcycling performance of able-bodied men in a well-controlled laboratory setting. The results indicate that HIIT improves upper body endurance capacity (peakVO_2_+22.2 ± 8.1%) and handcycling performance (peakPO +47.1 ± 20.7%) significantly more than MICT and CON. These findings suggest that HIIT sessions should be included in the training regimes of recreationally active and trained handcyclists to improve their upper body endurance performance. MICT also produced notable yet smaller improvements, by altering peakVO_2_ (+10.7 ± 12.9%) and peakPO (+32.2% ± 8.1). It thus seems that both HIIT and MICT, that are known to be effective in lower body exercise can be used to design upper body endurance training programs in handcycling.

## Author contributions

PS, FH, and LV contributed to conception and design of the work. PS conducted the experiment, analyzed the data and wrote the first draft. All authors (PS, FH, LV, and KR) were involved in further data analysis and drafting, and revised the manuscript critically for important intellectual content. All authors have approved the final version of the manuscript, agree to be accountable for all aspects of the work in ensuring that questions related to the accuracy or integrity of any part of the work are appropriately investigated and resolved, and all persons designated as authors qualify for authorship, and all those who qualify for authorship are listed.

### Conflict of interest statement

The authors declare that the research was conducted in the absence of any commercial or financial relationships that could be construed as a potential conflict of interest.
